# Free light-shape focusing in extreme-ultraviolet radiation with self-evolutionary photon sieves

**DOI:** 10.1038/s41598-024-51902-1

**Published:** 2024-01-19

**Authors:** Huaiyu Cui, Xiuping Zhang, You Li, Dongdi Zhao, Junyong Zhang, Yongpeng Zhao

**Affiliations:** 1 National Key Laboratory of Science and Technology On Tunable Laser, Harbin Institute of Technology, Harbin, China; 2 Key Laboratory of High Power Laser and Physics, Shanghai Institute of Optics and Fine Mechanics, Chinese Academy of Sciences, Shanghai, 201800 China

**Keywords:** Applied optics, Lasers, LEDs and light sources, Optical materials and structures, Optical techniques

## Abstract

Extreme-ultraviolet (EUV) radiation is a promising tool, not only for probing microscopic activities but also for processing nanoscale structures and performing high-resolution imaging. In this study, we demonstrate an innovative method to generate free light-shape focusing with self-evolutionary photon sieves under a single-shot coherent EUV laser; this includes vortex focus shaping, array focusing, and structured-light shaping. The results demonstrate that self-evolutionary photon sieves, consisting of a large number of specific pinholes fabricated on a piece of Si_3_N_4_ membrane, are capable of freely regulating an EUV light field, for which high-performance focusing elements are extremely lacking, let alone free light-shape focusing. Our proposed versatile photon sieves are a key breakthrough in focusing technology in the EUV region and pave the way for high-resolution soft X-ray microscopy, spectroscopy in materials science, shorter lithography, and attosecond metrology in next-generation synchrotron radiation and free-electron lasers.

## Introduction

Extreme ultraviolet (EUV) radiation sources are tools with significant potential in various advanced scientific fields, such as microscopic-activity probing^1^, nanoscale structure processing^2^, and high-resolution imaging^3^. Especially in high-resolution imaging, EUV imaging with high-order harmonic sources^4–6^ and EUV ptychography^7–9^ have made significant progress in recent years. Lensless diffractive-imaging techniques are well suited to EUV; they require spatially coherent beams and replace imaging elements in an optical system with a computerized phase-retrieval algorithm^10–12^.

Meanwhile, EUV radiation facilities based on different schemes, such as free-electron lasers^13^, laser-induced plasma radiation^14^, synchrotron radiation^15^, high-order harmonic generators^4–6,16^, and capillary-discharge lasers^17,18^, are under rapid development with higher output energy, shorter duration, and more diverse wavelengths. The lack of optical devices for light-shape focusing in the EUV region hinders their application in high-performance radiation facilities.

To date, several methods exist for focusing EUV radiation into a single point. The functions and characteristics of Schwarzschild mirrors coated by multiple layers (e.g., Sc/Si for 46.9 nm and Mo/Si for 13.5 nm) exhibit a relatively high reflectivity at normal incidence based on the Prague formula; they are more suitable for focusing EUV radiation into a tight focus^19,20^. Fresnel-zone plates (FZP) provide more flexible EUV light focusing to a certain extent^21^; however, the spatial resolution of FZP is on the order of the width of its outermost zone.

Recently, Ossiander et al. demonstrated that a metalens could efficiently focus vacuum-guided EUV light^22^. This method has the potential for diverse light shaping in the EUV region, whereas EUV metalenses require ultra-high-precision machining, which approaches the limit of electron-beam etching. Photon sieves (PS), proposed by Kipp et al. in 2001, can focus EUV and soft X-ray radiation with suppressed side lobes, compared to traditional FZP^23^. Despite their significant potential for diverse light shaping in the EUV region^24^, to the best of our knowledge, photon sieves have never been experimentally applied to EUV radiation for free light shaping.

In this study, self-evolutionary photon sieves were developed and applied to a capillary-discharge extreme-ultraviolet laser for free light-shape focusing, including vortex focus shaping, array focusing, and structured-light shaping. The results demonstrate the latent capability of the improved photon sieves in the EUV region and open up an infinite number of applications for EUV radiation.

### Design principle of self-evolutionary photon sieves

The functional limitations of traditional photon sieves are primarily due to their isotropic structures. If the structure is asymmetric and anisotropic, then the monofocal distribution no longer exists. For an expected field distribution, we can use a genetic algorithm (GA) to optimize the structure of self-evolutionary photon sieves^25–27^.

Consider the trifocal PS as an example. The trifocal spots are distributed along the light-propagation direction corresponding to a symmetric structure; thus, a traditional PS can be chosen as the initial optical structure. All pinholes are divided into two groups, based on whether each pinhole is distributed on a transparent or opaque ring. Each subunit is constructed by randomly selecting pinholes in either group with equal probability, and is encoded as the initial population.

Subsequently, the objective-function value can be used as the fitness function for each individual. Thus, the genetic operator, including the selection, crossover, and mutation operators, is applied to the encoded population, and a new population is created. Finally, if the optical structure corresponding to the new population produces the expected diffraction patterns, the optimization is stopped. Otherwise, the optimization program repeats the aforementioned steps until the structure satisfies the requirements for diffraction-pattern.

**Figure 1:**
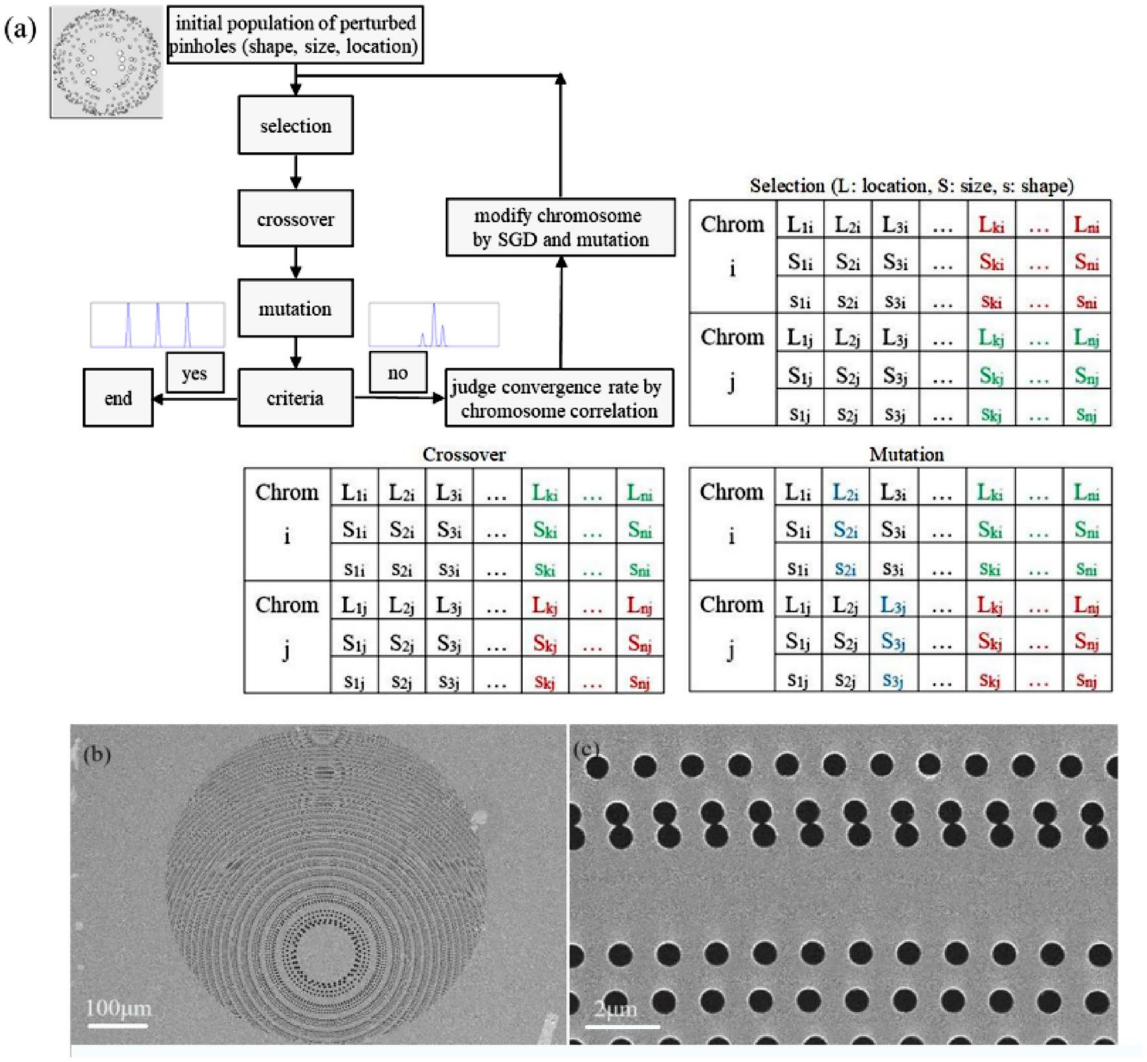
(**a**) Design flowchart of a modified spiral PS using a genetic algorithm (GA) and stochastic gradient descent (SGD), (**b**) tri-focal PS detected by scanning electron microscope (SEM), (**c**) outermost ring.

The table in Fig. 1a lists the three main operators of the GA. The selection operation is used to generate parent chromosomes and prepare the chromosomes required for the next operation. Spiral-PS information is encoded into one chromosome containing N genes, each of which is labeled by the pinhole location (L_n_), size (S_n_), and shape (s_n_). The principle of the crossover operation is that genetic segments in one chromosome are randomly selected and exchanged with those of another chromosome at the corresponding mated position. Mutation is a key operation that determines the convergence rate of the GA. In this study, the mutation rate was chosen randomly between 0.1 and 0.2.

Following the completion of the aforementioned steps, we obtain the parameters of the modified spiral PS, such as the pinhole location, size, and shape. The size and distribution of the focal spots are considered as fitness functions. Figure 1b,c illustrate photographs of the trifocal PS detected by scanning-electron microscopy (SEM). The diameter of the pinhole at the outermost ring is approximately 400 nm, which is much larger than the manufacturing limit, indicating the practicability of the PS for free light-shape focusing in the EUV region.

To obtain other types of focal spots, only the initial population needs to be replaced; the object function is also modified according to the requirements under different scenarios. In addition, to avoid iterative stagnation in the optimization process, a hybrid optimization algorithm is typically adopted to accelerate the convergence rate and improve the accuracy of the structure.

Generally, a traditional PS has millions of transparent pinholes. The shape, size, and location of each pinhole can be controlled as required to further enhance the design freedom. Infinite degrees of freedom imply that the required field must be constructed theoretically, regardless of its complexity.

**Figure 2:**
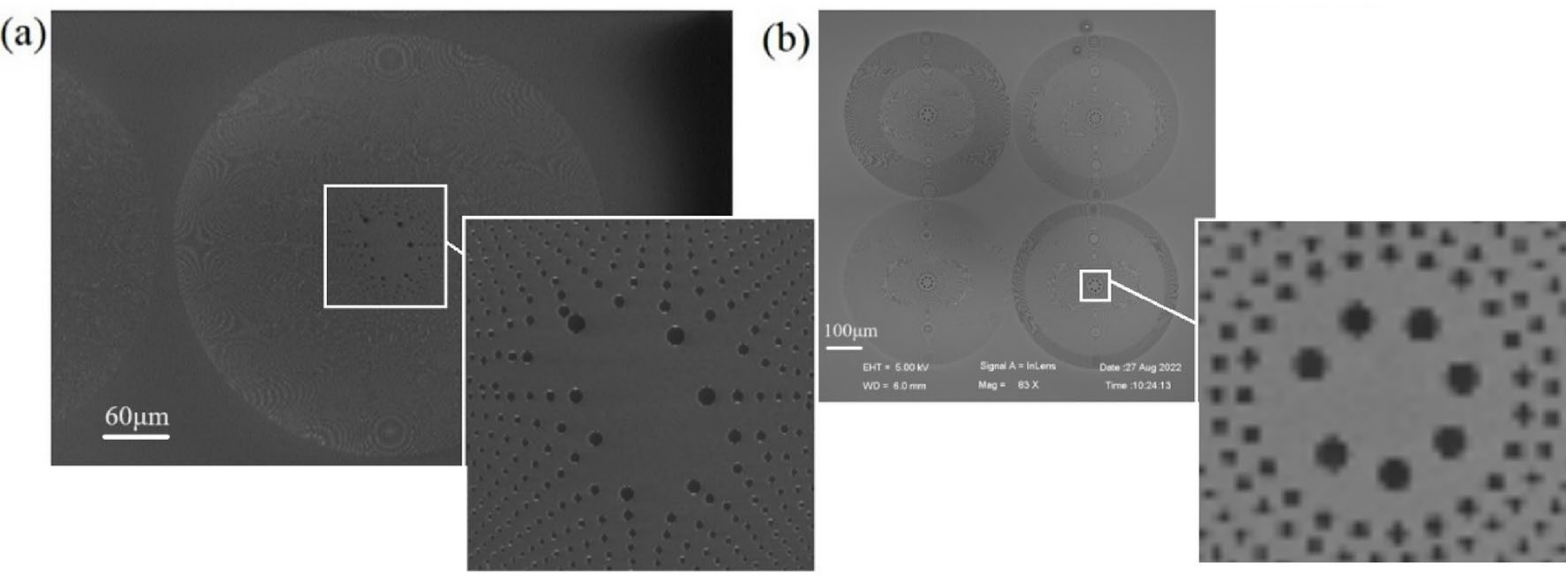
SEM photograph of (**a**) spiral PS splitter and (**b**) light-shaping PS.

In contrast to the solid focal spots, two other complex structured lights are indicated, according to the methods mentioned earlier. Figure 2a illustrates an SEM photograph of the spiral PS splitter, in which the orbital angular momentum is encoded into the structure of a traditional PS. Figure 2b presents a group of light-shaping PSs that can generate multiplanar structured light consisting of doughnut and dot-doughnut focusing.

### Experiments with self-evolutionary photon sieves

In our experiment, the EUV radiation is provided by a capillary-discharge 46.9-nm laser operating in single-shot mode with 0.5 mJ of output energy and a 1.6-ns duration^18,28,29^. The laser is generated using a Z-pinched Ar^8+^ plasma column excited by a fast current with a ~ 25-kA amplitude. The PSs are positioned along the beam path and fixed to the bottom of the vacuum chamber. Transparent pinholes are directly fabricated on the Si_3_N_4_ membrane using a one-step pattern-transfer process^30^.

The PS patterns were first defined on a ZEP520A resist using electron-beam lithography (EBL). The 100-nm-thick self-supporting Si_3_N_4_ membrane substrate was fabricated from a silicon–nitride wafer via potassium-hydroxide (KOH) etching. The e-beam resist (ZEP520A) was spin-coated onto the Si_3_N_4_ membrane substrate. Following e-beam exposure, the samples were developed in a mixture of methyl isobutyl ketone (MIBK) and isopropyl alcohol (IPA) and rinsed in IPA. Using the defined ZEP520A as the etching mask, the freestanding sieves were prepared using fluorine-based reactive ion etching (RIE) to etch the Si_3_N_4_ layer with the transparent pinholes.

**Figure 3:**
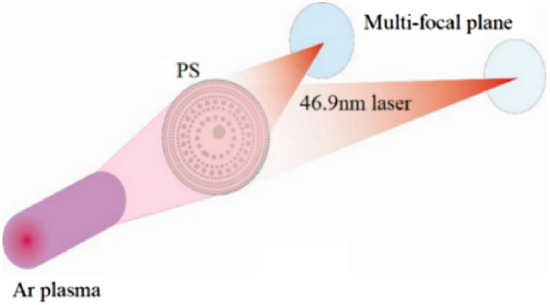
Experimental setup in which self-evolutionary photon sieves are illuminated by a 46.9-nm laser.

The recording medium, polymethyl methacrylate (PMMA) (950,000 molecular weight), coated on a silicon wafer is used to record the focal spots. The experimental setup is illustrated in Fig. 3. The PMMA target is fixed on a two-dimensional stage to perform a z-scan along the beam axis. Considering the difference between the focal spots of the PSs, the energy of the single-pulse laser that deposits on the PMMA surface is less than 1 μJ. Following a single-shot exposure, the PMMA targets are developed and steeped in a solution for a flexible time to obtain a measurable shaped light field. The processed PMMA targets are detected using atomic-force microscopy (AFM).

## Vortex focus shaping

**Figure 4:**
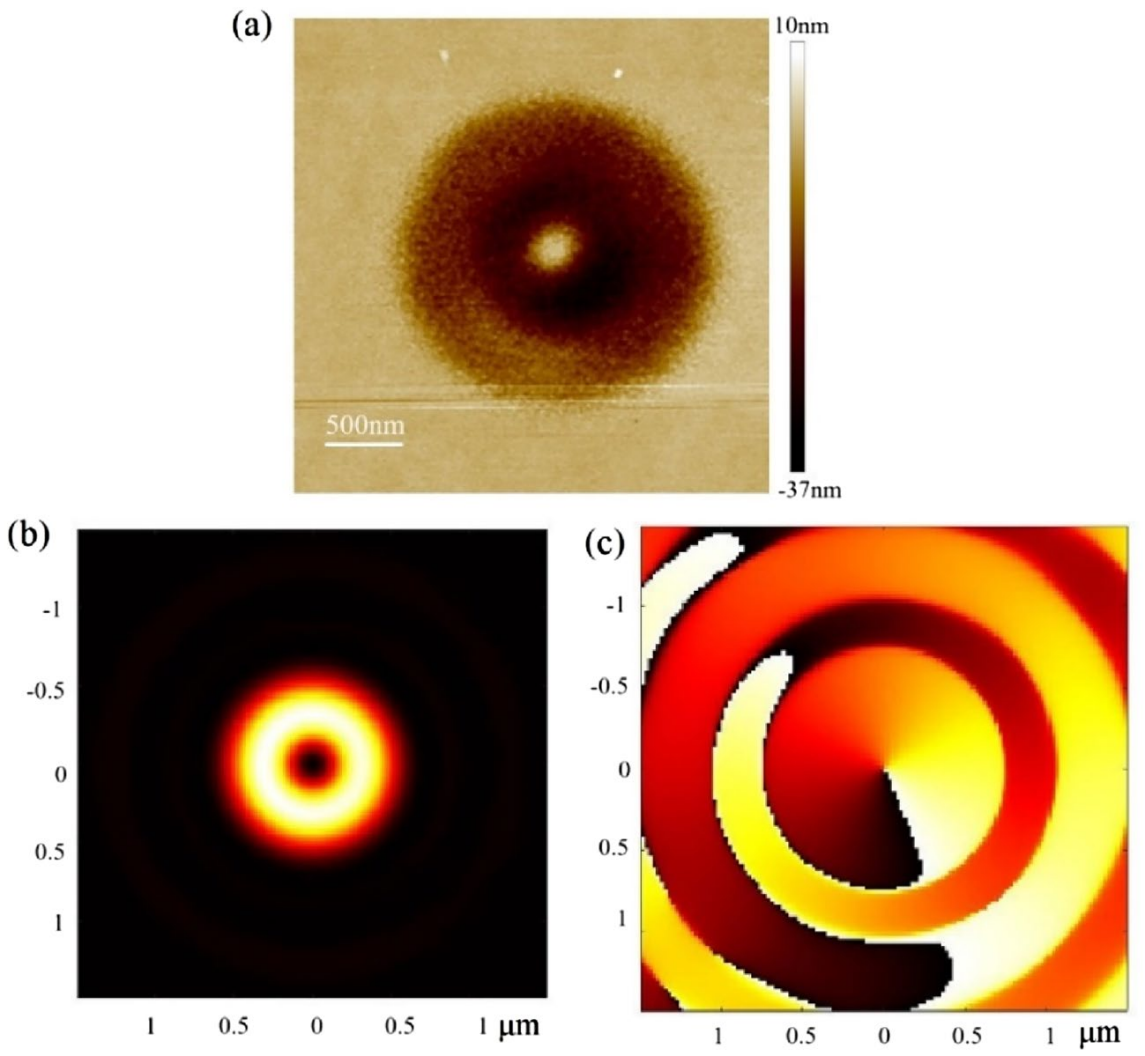
Vortex focusing. (**a**) EUV spot recorded by PMMA at the focal plane. (**b**) Simulated energy distribution at the focal plane. (**c**) Simulated phase distribution at the focal plane.

Optical vortices are widely used in numerous fields, such as optical tweezers, trapping, cryptography, communication, and nonlinear and quantum optics. Figure 4 illustrates the focused vortex EUV light generated by a spiral PS with a 291-μm diameter and 2.5-mm focal length, in which a vortex-shaped phase and doughnut-shaped energy are distributed at the focal plane. The orbital angular momentum derives from the helical phase structure of the spiral PS. The topological charge of the vortex focal spot equals one, as illustrated in Fig. 4c. When a spiral PS is chosen as the initial structure, a splitting vortex focal spot can be generated after optimization.

**Figure 5:**
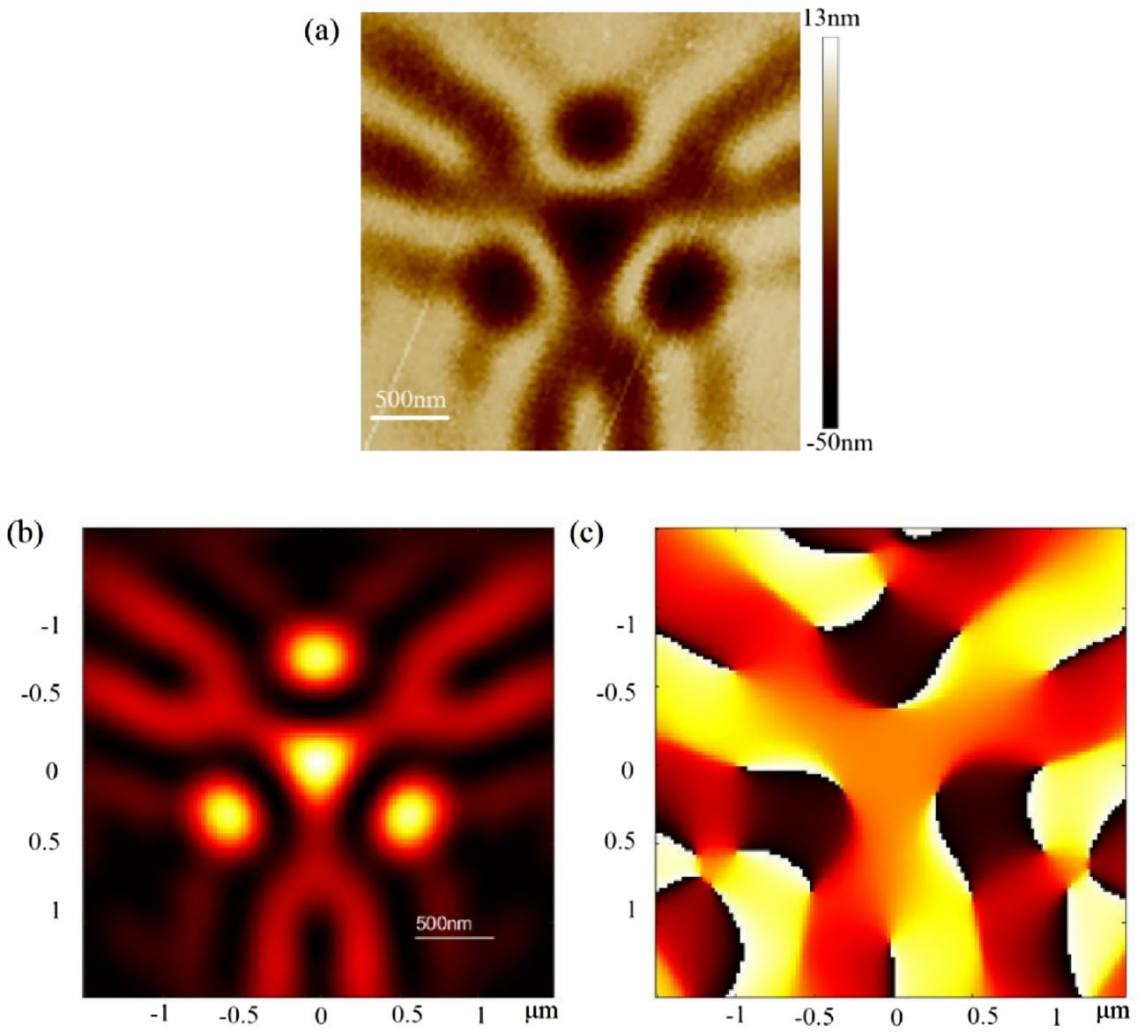
Splitting vortex focusing. (**a**) EUV spot recorded by PMMA at the focal plane. (**b**) Simulated energy distribution at the focal plane. (**c**) Simulated phase distribution at the focal plane.

Figure 5 illustrates the splitting vortex focal spot and the theoretical simulation. This indicates that the shape of the modulatory EUV laser spot agrees well with the simulation, even in the side lobe. Figure 5c shows the phase distribution of the EUV focal spot. The vortex-focusing experiment indicates that the self-evolutionary PS can operate EUV light in the form of an anamorphic spiral. It is not difficult to imagine that PSs can manipulate EUV light with diverse energy and phase distributions, which has inestimable application prospects in interferometry and diffractometry.

### Structured-light shaping

**Figure 6:**
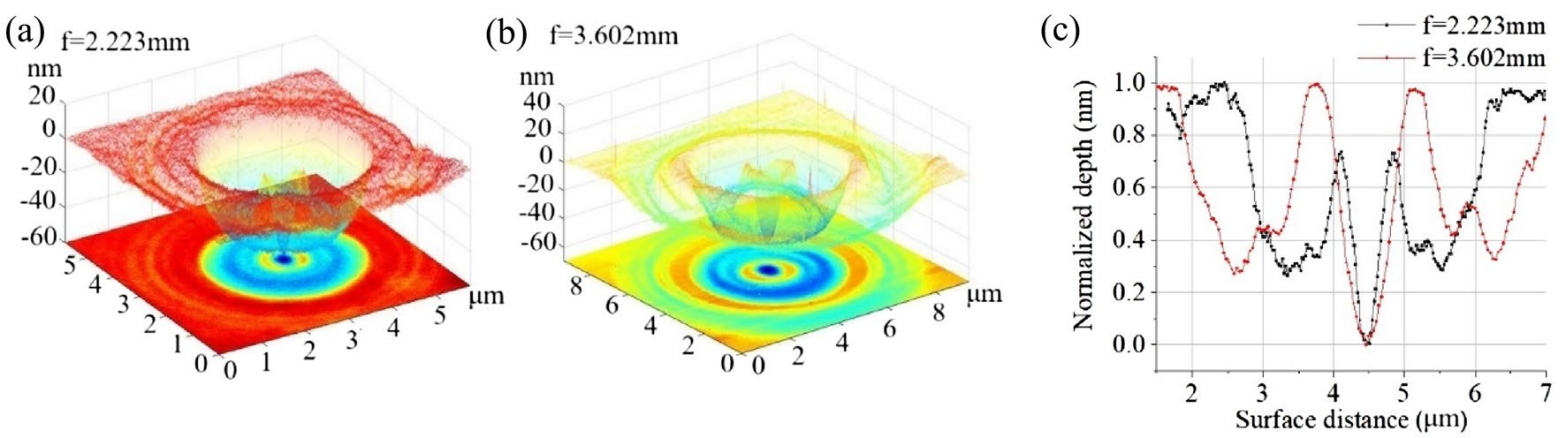
Focusing EUV shaped into one ring with a peak in the center on a bi-focal plane. (**a**) Recorded focused spot at f = 2.223 mm. (**b**) Recorded focused spot at f = 3.602 mm. (**c**) Cross section of the focal spots at two focal planes.

PSs can be designed to generate structured EUV light. In this section, the PS splits the incident laser beam into two focal planes with focal lengths of 2.223 and 3.602 mm. Subsequently, at each focal plane, the laser is shaped into a given field distribution consisting of one ring and one peak at the center, as illustrated in Fig. 6.

The EUV light fields are recorded by PMMA. Similar to traditional PSs, self-evolutionary PSs can suppress the side lobe by several orders of magnitude. In addition, the diameters of the structured EUV-laser focal spots increase with the focal length. The outmost diameter of the focal spot in Fig. 6a is 3.585 μm, while that of the focal spot in Fig. 6b is 5.294 μm. The fundamental reason for this phenomenon is that different numerical apertures (NAs) correspond to different focal lengths.

**Figure 7:**
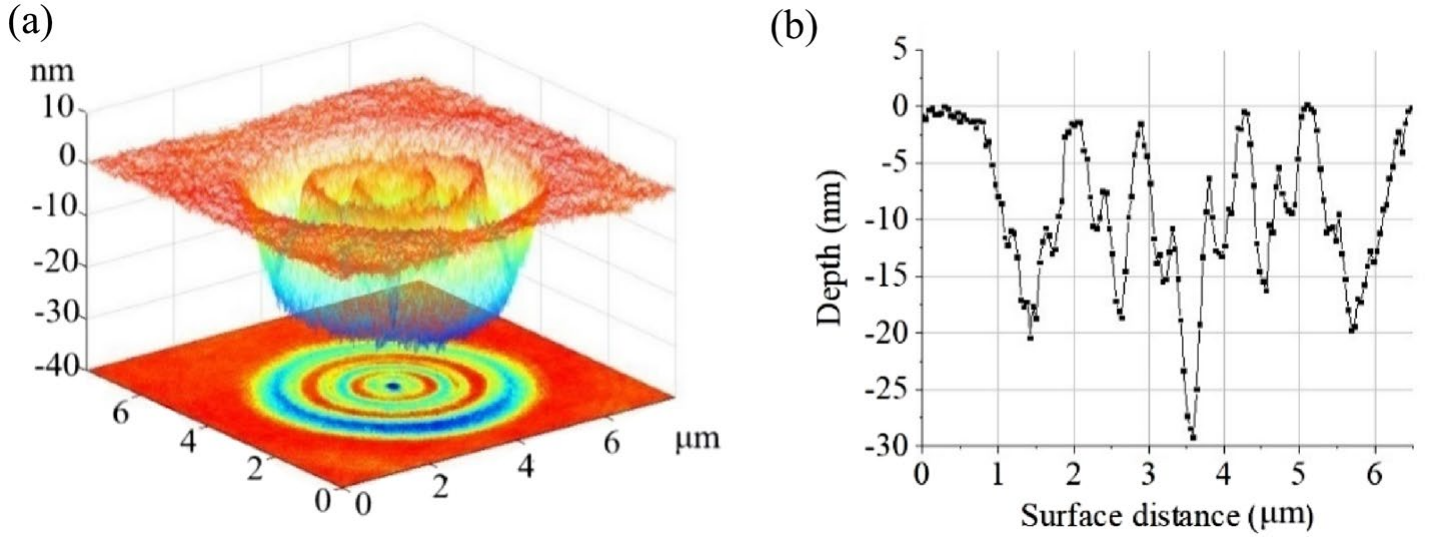
Complex-shaped EUV laser focused at a focal length of 2.223 mm. (**a**) Recorded focused spot. (**b**) Cross section of the focal spot.

The results in Fig. 6 indicate that the EUV light can be split and shaped into the expected field with a single PS. Moreover, the self-evolutionary PS can shape EUV light into more complex structures. Figure 7 illustrates a more complex EUV light structure focused at a focal length of 2.223 mm. In general, the self-evolutionary PS can easily split the EUV light into numerous more focal planes, as required for the designed field distribution, provided the splitting light at each focal plane can trigger the recording medium. Structured EUV light focused on multiple planes has significant potential for use in high-resolution array imaging, structured nanometer focusing, and other related research fields.

### Array focusing and phase-shift imaging

**Figure 8:**
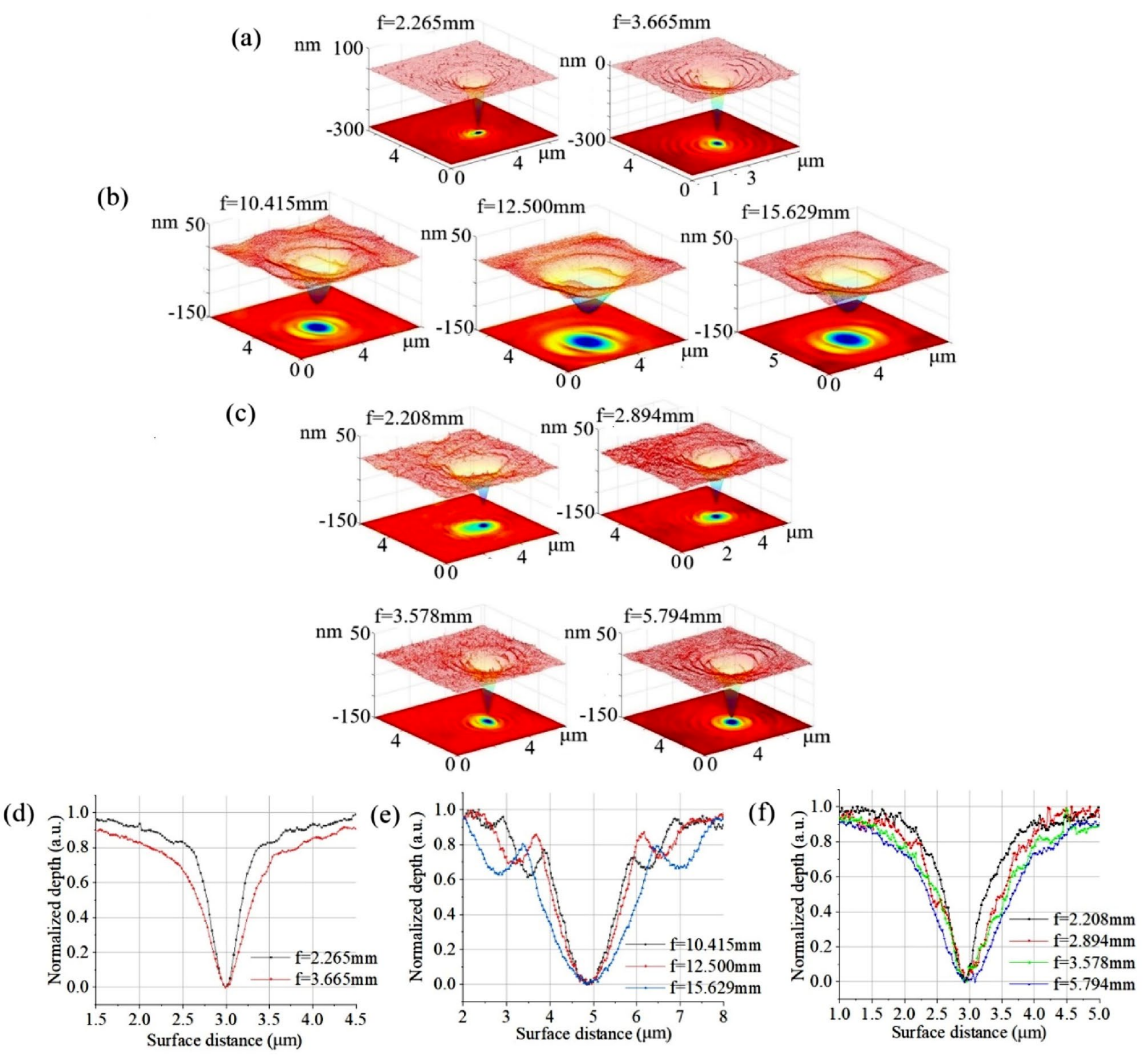
Array focusing recorded by PMMA, generated by (**a**) bi-focal, (**b**) tri-focal, and (**c**) quad-focal PSs, and the cross-section of the focal spots generated by (**d**) bi-focal, (**e**) tri-focal, and (**f**) quad-focal PSs.

Bi-focal, tri-focal, and quad-focal PSs, with diameters of 445.4 μm, 838.9 μm, and 439.9 μm, respectively, are used to focus the EUV laser. Following a single-shot exposure to the 46.9-nm laser, focal spots at multiple planes were recorded by PMMA and measured by AFM. Figure 8 illustrates the profile and cross-section of the focal spots generated by the bi-focal PS with focal lengths of 2.265 mm and 3.665 mm, tri-focal PS with focal lengths of 10.415, 12.500, and 15.629 mm, and quad-focal PS with focal lengths of 2.208, 2.894, 3.578, and 5.794 mm. A clear focal spot with a good profile was recorded in each focal plane. For the same PS diameter, as shown in Fig. 8d–f, a larger focal spot corresponds to a longer focal length, which is consistent with the theoretical analysis.

With an array-focusing PS, EUV lasers can be split and focused freely in a three-dimensional space, and the multifocal lengths can be corrected by modifying both their positions and the distribution of the transparent pinholes. This is useful for self-interference holography or phase recovery in the phase-imaging field because the phase difference is fixed between two focal planes, and multiple diffraction patterns can be achieved by a single exposure. Below, we discuss array focusing for fast and high-precision imaging in the optical band.

**Figure 9:**
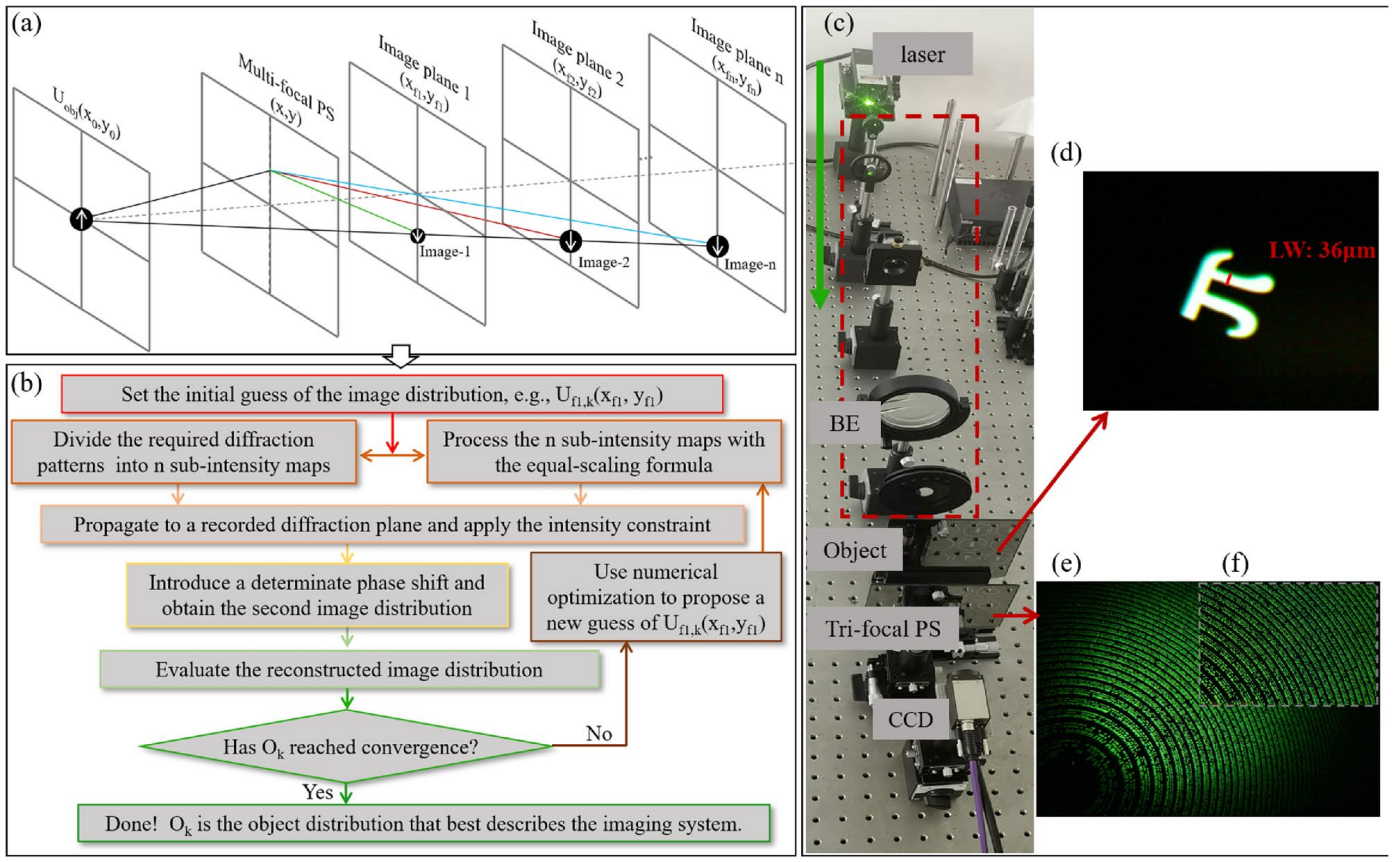
(**a**) Principle of the single-shot PSIPD. (**b**) Flowchart of the PSIPD algorithm. (**c**) Experimental setup, beam expander (BE). (**d**) Test object, line-width (LW). (**e**–**f**) Micrograph of the multi-focal phase-shifting PSs.

For traditional phase diversity (PD)^31–33^, the phase difference between two diffractograms must be known. Unlike monofocal lenses, multifocal PSs can produce more than one image with a known phase difference. In this case, phase-shift image phase diversity (PSIPD) can be used to effectively recover the quantitative phase in the EUV and soft X-ray regimes. The major difference between PD and PSIPD is that the phase difference in PSIPD is only determined by the PS parameters, rather than by the slight axial movement in the PD, as shown in Fig. 9a. A multifocal PS can produce multiple images. The image distribution in each image plane can be written as

1 $${E}_{out\_j}\left(X,Y\right)\propto {e}^{-i\left[\frac{\pi \left({X}^{2}+{Y}^{2}\right)}{\left(\uplambda M{f}_{j}\right)}\right]}{E}_{i{n}_{j}}\left(\frac{X}{M},\frac{Y}{M}\right),$$

where the subscript *j* denotes the *j*th image, *E*_*in_j*_(.) and *E*_*out_j*_(.) denote the test object and its image, respectively, *M* is the lateral magnification, and λ denotes the incident wavelength.

Figure 9b illustrates the flowchart of the PSIPD algorithm. Each image was processed according to the scaling factor. The initial guess U_f1, k_(x_f1_, y_f1_) is the square root of the random intensity distribution multiplied by an arbitrary constant phase, where subscript *k* represents the *k*th iteration and (x_f1_, y_f1_) is the image plane. Subsequently, U_f1, k_(x_f_, y_f_) propagates to the recorded diffraction plane and the diffraction pattern is denoted as U_obj, k_(x_1_, y_1_). The amplitude is replaced with the square root of the measured intensity. The standard iteration from the first diffraction plane to the last is processed sequentially and finally backpropagates to the guess plane U^’^_f1, k_(x_f1_, y_f1_).

Based on Eq. (1), each image plane introduces a determinate phase shift. The new guess is scaled to obtain another image and then propagates its own diffraction pattern. Each image operation is the same as that of round 1; thus, a new image can be obtained. Following the scaling operation, the number of images is equal to the number of focal lengths. At this moment, the guess image is the mean of these images. Each diffraction pattern is propagated to its own image plane.

The phase difference between any two image planes is close to the value obtained using Eq. (1), which corresponds to the convergence condition. Once the image distribution is obtained, the test object can be directly obtained from the conjugation relationship between the object and image space^34–37^.

A 532-nm central wavelength laser beam illuminates the test object with the 36-μm line-width (as shown in Fig. 9d). Subsequently, it passes through the tri-focal PSs (as shown in Fig. 9e–f) with focal lengths of 60 mm, 48 mm, and 40 mm, in which approximately 486-thousand transparent pinholes are fabricated in a chrome plate.

**Figure 10:**
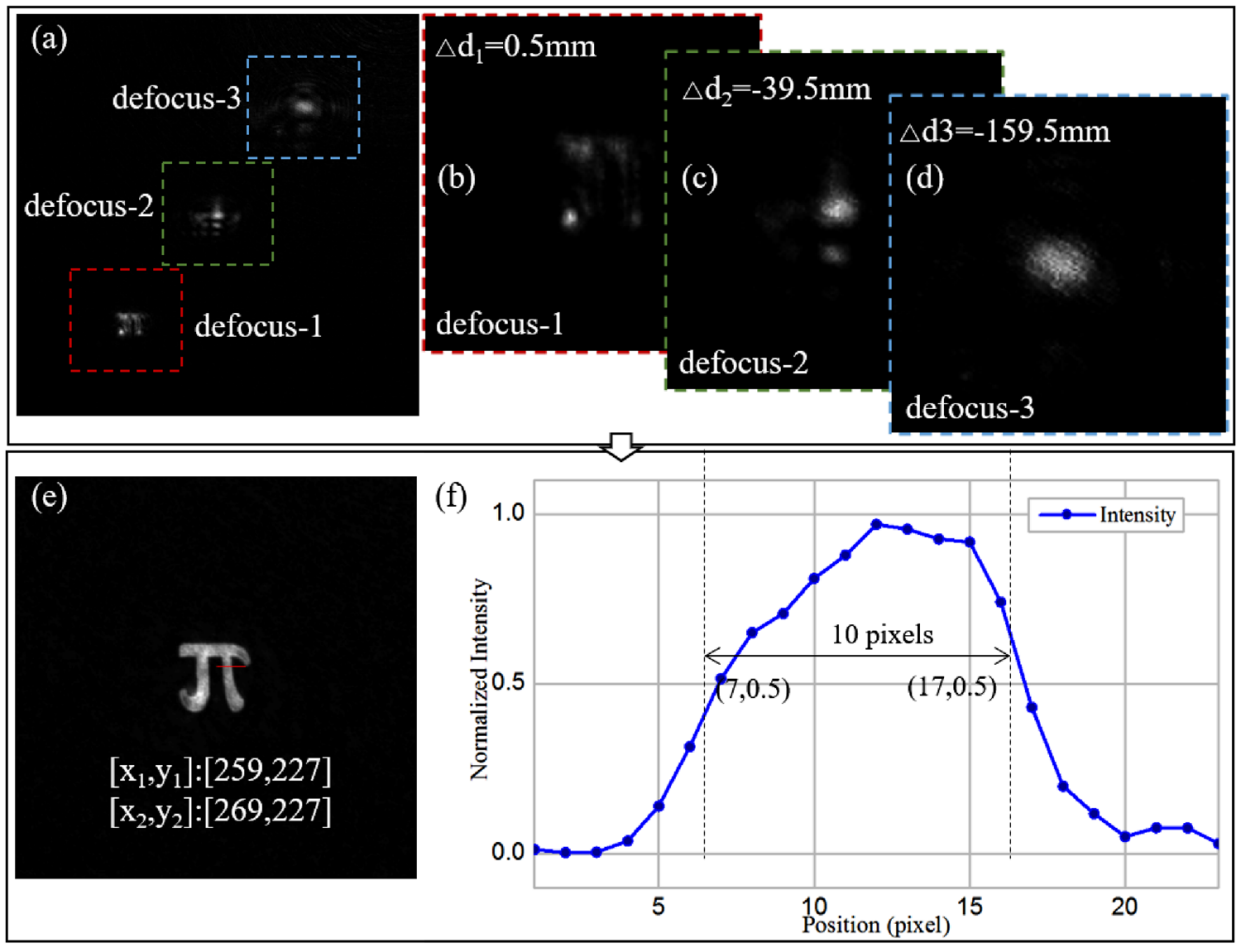
Experimental results of a “π” fabricated on a chrome plate. (**a**–**d**) Diffraction patterns, (**e**–**f**) recovered results.

In the experiment, we used the defocused intensity distribution of an amplitude-type object to recover the measured distribution, as shown in Fig. 10. The results of recovering the phase objects by the PSIPD method can be found in reference^38^. The defocused diffractograms are recorded by a CMOS detector (4096 × 3000 resolution, 3.45-μm pitch size). Figure 10a–d show the recorded diffractograms in a single exposure, which includes three defocused patterns in the same recording plane. Three independent patterns were extracted, and the retrieved images were solved clearly, as shown in Fig. 10e. The x-axis line-width is 10 pixels, as shown in Fig. 10f; thus, the line-width of the reconstructed image is about 34.5 μm, which is in good agreement with the theoretical value of 36 µm. The experimental results prove the validity of the proposed single-shot PSIPD with multifocal PSs.

Note that multifocal PSs can break the limit of the imprecise distance between any two recording planes to improve recovery accuracy. A weak signal should be recorded near the focal plane, whereas a strong signal can be recorded far from the focal plane to improve the signal-to-noise ratio. The proposed PSIPD with amplitude-only PSs is useful for high-resolution EUV imaging.

## Conclusion

In summary, EUV light was demonstrated to be free light-shape focused by single self-evolutionary PSs with a capillary-discharge 46.9-nm laser in terms of vortex focus shaping, array focusing, and structured-light shaping. The strong medium absorption led to difficult focusing and imaging in the EUV and soft X-ray region; however, the emergence of freestanding self-evolutionary PSs with amplitude-only modulation successfully filled the gap between EUV and X-ray high-resolution focusing and imaging. A series of experiments was carried out successfully, and the results of this work provided a practical method for modulating EUV radiation, which means that light shaping in the EUV domain is no longer a difficult challenge.

## Data Availability

The datasets used and/or analyzed in the current study are available from the corresponding author upon reasonable request.
